# Microbial aetiology of brain abscess in a UK cohort: Prominent role of Streptococcus intermedius

**DOI:** 10.1016/j.jinf.2020.03.011

**Published:** 2020-06

**Authors:** Christopher A Darlow, Nicholas McGlashan, Richard Kerr, Sarah Oakley, Pieter Pretorius, Nicola Jones, Philippa C Matthews

**Affiliations:** a Institute of Translational Medicine, University of Liverpool, Ashton St, Liverpool, L69 3GE, UK; b Department of Infectious Diseases and Microbiology, Oxford University Hospitals NHS Foundation Trust, John Radcliffe Hospital, Headington, Oxford OX3 9DU, UK; c Department of Neuroradiology, Oxford University Hospitals NHS Foundation Trust, John Radcliffe Hospital, Headington, Oxford OX3 9DU, UK; d Department of Neurosurgery, Oxford University Hospitals NHS Foundation Trust, John Radcliffe Hospital, Headington, Oxford OX3 9DU, UK; e Nuffield Department of Medicine, University of Oxford, Medawar Building for Pathogen Research, South Parks Road, Oxford OX1 3SY, UK; f NIHR Oxford British Research Council (BRC), John Radcliffe Hospital, Headington, Oxford OX3 9DU, UK

**Keywords:** Brain abscess, Aetiology, Microbiology, Epidemiology, Prevalence, Streptococci, Streptococcus milleri, Imaging, antibiotics

## Abstract

**Background:**

Brain abscess is an uncommon condition, but carries high mortality. Current treatment guidelines are based on limited data. Surveillance of clinical, radiological and microbiology data is important to inform patient stratification, interventions, and antimicrobial stewardship.

**Methods:**

We undertook a retrospective, observational study of patients with brain abscess, based on hospital coding, in a UK tertiary referral teaching hospital. We reviewed imaging data, laboratory microbiology, and antibiotic prescriptions.

**Results:**

Over a 47 month period, we identified 47 adults with bacterial brain abscess (77% male, median age 47 years). Most of the abscesses were solitary frontal or parietal lesions. A microbiological diagnosis was secured in 39/47 (83%) of cases, among which the majority were of the *Streptococcus milleri* group (27/39; 69%), with a predominance of *Streptococcus intermedius* (19/27; 70%). Patients received a median of 6 weeks of intravenous antibiotics (most commonly ceftriaxone), with variable oral follow-on regimens. Ten patients (21%) died, up to 146 days after diagnosis. Mortality was significantly associated with increasing age, multiple abscesses, immunosuppression and the presence of an underlying cardiac anomaly.

**Conclusion:**

Our data suggest that there has been a shift away from staphylococcal brain abscesses, towards *S. intermedius* as a dominant pathogen. In our setting, empiric current first line therapy with ceftriaxone remains appropriate on microbiological grounds and narrower spectrum therapy may sometimes be justified. Mortality of this condition remains high among patients with comorbidity. Prospective studies are required to inform optimum dose, route and duration of antimicrobial therapy.

## Highlights

• A microbiological diagnosis can currently be secured for >85% of brain abscesses.
• The predominant organism is *Streptococcus intermedius*.
• Patients typically receive six weeks of intravenous ceftriaxone (± metronidazole).
• Mortality of brain abscesses remains high at >20%.

## Introduction

Brain abscesses are a focal infection characterised by a walled-off collection of pus within the brain parenchyma. They may arise spontaneously, or as a result of specific risk factors including intravenous drug use, congenital cardiac defects, infective endocarditis, immunosuppression, or a contiguous focus of infection spreading directly to the adjacent central nervous system (CNS), for example from dental, sinus, and middle or inner ear infection.^1^, ^2^, ^3^, ^4^, ^5^ Previous reports suggest that brain abscesses predominantly occur in males (accounting for around 70% of cases) and affect young adults.^6^^,^^7^

Brain abscesses are usually caused by bacteria, although can also be caused by other pathogens including fungi and protozoa.^4^ Prior to 1960, staphylococcal infection was the commonest reported cause, accounting for over a third of cases, with streptococci accounting for around a further 30%.^6^ However, in recent decades, the incidence of staphylococcal brain abscesses has been falling, with streptococci increasingly dominating.^6^^,^^7^ Organisms in the *Streptococcus milleri* group are frequent commensals of the oropharynx and gastrointestinal tract. They are well recognised for a propensity to cause abscesses, and are a recognised cause of central nervous system infections.^8^^,^^9^ The *S. milleri* group includes *S. constellatus, S. anginosus* and *S. intermedius*, and can also be classified by phenotypic identification as ‘microaerophilic streptococci’, reflecting their optimum conditions for growth in partial pressures of oxygen that are lower than atmospheric O_2._^8^^,^^10^ Pathogenesis is mediated by β-haemolysis and production of extracellular enzymes such as hyaluronidase and DNAase.^8^^,^^11^, ^12^, ^13^

Anaerobes are also described as important contributors to brain abscesses, and polymicrobial aetiology has been described in around a quarter of cases. However, evidence for the presence of anerobes is limited, as studies before 2010 report identification of a pathogen in only approximately 60% of cases,^14^^,^^15^ and even with improved approaches to diagnosis, sensitivity of laboratory diagnosis may be limited for anaerobic organisms. With modern stereotactic neurosurgical sampling methods and advances in molecular diagnostics, there is scope for improving rates of identification of specific pathogens, up to ∼80% in the more recent literature,^7^ providing an opportunity to review approaches to antimicrobial therapy.

There are no recent unifying international or national guidelines for the management of bacterial brain abscesses. An ‘Infection in Neurosurgery Working Party’ published antibiotic recommendations on behalf of the British Society of Antimicrobial Chemotherapy in 2000.^16^ However, these recommendations were based on limited published data, and are not universally adopted in clinical practice. Other recommendations are made elsewhere in the existing literature, including the need to rule out other causes of space-occupying lesions (such as primary or metastatic malignancy), and additional investigations including an HIV test and consideration of cardiac echocardiography.^4^^,^^5^^,^^17^ The recommended empirical antimicrobial treatment for brain abscesses is often intravenous ceftriaxone or cefotaxime,^5^^,^^18^ which cover the likely gram-positive causes, as well as providing coverage for the less common gram negative organisms, and offering sufficient central nervous system penetration. Adjunctive metronidazole may also be recommended to cover anaerobic organisms.^16^ Despite the microaerophilic nature of *S milleri*, metronidazole is largely ineffective against this group.^19^

However, the suggested choice, duration and route of antibiotic therapy is based on scarce evidence.^20^ In the current era of increasing antimicrobial resistance, careful scrutiny of laboratory data is required to ensure that empiric treatment regimens are sufficiently broad to cover the majority of likely pathogens, while at the same time considering antimicrobial stewardship to limit unnecessary exposure to broad-spectrum agents. Furthermore, new evidence for endocarditis and for musculoskeletal infection suggests that oral antibiotics are non-inferior to parenteral therapy in some deep-seated infections.^21^^,^^22^ At present there are no such data to inform the management of CNS infection, but the route of therapy remains an important question to be addressed.

Given this changing landscape of antimicrobial therapy, the paucity of epidemiological data, and the limited guidelines for brain abscesses, we set out to collect clinical and laboratory data from a large UK teaching hospital to examine the epidemiological and microbiological trends of bacterial brain abscesses. We specifically undertook to investigate: i) Evidence for changes in the epidemiology or microbiology of brain abscesses over time; ii) The extent to which antimicrobial prescribing was adherent to local guidelines; iii) The extent to which current antimicrobial management strategies are appropriate to cover the organisms identified, considering both empirical and directed therapy; iv) Determination of any host factors that are associated with specific outcomes, in order to identify high risk cases, with implications for prognosis, monitoring and interventions.

## Methods

### Data collection

We collected data retrospectively from the electronic patient records of Oxford University Hospitals (OUH) NHS Foundation Trust, a large teaching hospital trust in the Thames Valley region of the UK and a tertiary referral centre for neurosurgery. We identified patients using coding data (any patients coded with ‘G06.0′, the ICD code for ‘Intracranial abscess and granuloma’) admitted between 1st January 2013 and 1st December 2016. We screened individual records to remove paediatric cases, erroneously coded cases and those with non-bacterial causes (e.g. *Toxoplasma gondii*), and then reviewed electronic notes and results, extracting the relevant data. The study was undertaken as a registered audit to determine the quality of management of brain abscesses. Approval for this was given internally via the OUH audit management team and we collected and stored data in accordance with relevant governance standards. All data were anonymised prior to analysis.

### Radiology data

Brain abscesses in our cohort were typically diagnosed by computer topography (CT) scan, in some cases followed by magnetic resonance imaging (MRI) to delineate further. We collected radiology data for the definitive pre-treatment scan only, as subsequent sequential scans varied according to clinical need. For the purposes of data collection for our study, all scans were retrospectively reviewed by two independent radiologists, blinded to all other clinical and microbiological data. To calculate the size of an abscess in cross-section, we assumed an elliptical area (area (mm^3^) = 0.5 x maximum width (mm) x 0.5 x maximum length (mm) x π).

### Laboratory diagnosis

Samples collected from a brain abscess would typically be transported to the microbiology laboratory on an urgent basis, and culture plates are set up promptly on receipt in the laboratory by Biomedical Scientists staffing a 24-hour service. Microbiology diagnostic work was undertaken in our ISO approved clinical diagnostic laboratory. Methods were based on National Standard Methods (PHE).^23^ In brief, samples from brain abscesses were handled as follows: a Gram stain was undertaken and the sample was inoculated into (i) cooked meat enrichment broth (extended incubation for 10 days in aerobic conditions), (ii) blood, chocolate and MacKonkey agar incubated in 5% CO_2_, (iii) blood, NAT and neomycin agar (in anaerobic conditions with metronidazole discs). All were incubated at 35–37 °C. Growth on any plate was followed up by identification using by mass spectrometry (Maldi-TOF). Antibiotic susceptibility was determined on the automated Phoenix platform (Becton Dickinson). Examination for additional specific pathogens would be undertaken at the request of the clinical team or based on attributes of the patient's background or presenting features (e.g. mycobacteria, nocardia, fungi, acanthamoeba).

For streptococci, susceptibility to penicillin was further investigated by determination of the minimum inhibitory concentration (MIC) based on the breakpoint defined by the European Committee on Antimicrobial Susceptibility Testing (EUCAST) using both Phoenix and an MIC strip (ETEST; Biomérieux); the breakpoints are ≤0.25 mg/L for benzylpenicillin and ≤0.5 mg/L for amoxicillin.^24^

Blood cultures were inoculated into BACTEC bottles under aseptic technique and incubated on the Becton Dickinson BACTEC FX system, using a standard operating procedure based on SMI B 37: ‘investigation of blood cultures’.^25^

### Antimicrobial therapy guidelines

We audited each case for adherence to local antimicrobial guidelines, which are accessible to clinical staff via intranet and the smartphone app ‘Microguide’ (http://www.microguide.eu/). For bacterial brain abscesses, antimicrobial advice in our setting is as follows:

• Urgent biopsy with collection of specimens for culture is recommended in most cases;
• Empiric choice of antimicrobial therapy is ceftriaxone (2 g iv bd) and metronidazole (400 mg tds po);
• For empiric regimens, the cephalosporin should be substituted with ciprofloxacin in patients with beta-lactam allergy, and vancomycin should be added in cases known to be colonised or infected with meticillin resistant *Staphylococcus aureus*;
• For patients at risk of immunosuppression, empiric therapy may be amended to broaden spectrum of cover;
• Initial empiric treatment should be amended according to culture and susceptibility data;
• The microbiology/infectious diseases team should be consulted for clinical review and advice;
• Treatment is recommended for 4–8 weeks.

### Data regarding surgical and medical management

A limited dataset regarding surgical intervention and antimicrobial therapy was collected from the electronic patient record. Antimicrobial therapy was monitored and modified under supervision of the Infectious Diseases / Microbiology team according to the clinical context and microbiology results. For patients well enough to be discharged but requiring ongoing parenteral therapy, the full course of antibiotics was administered under the supervision of the Oxford outpatient antimicrobial therapy (OPAT) team via a peripherally inserted central catheter (PICC).^26^

### Statistical methods

Descriptive statistics of the data was collated using Microsoft Excel and SPSS software packages. Univariate associations were explored using Fisher's Exact Test, one-way ANOVA or Kruskal-Wallis statistical tests, depending on the modality and distribution of the data.

## Results

### Brain abscess patients presented to our service at an average rate of 1 per month and were predominantly male

Over the study time period of 47 months, 74 patients were coded by the hospital as G06.0. Of these, 47 were aged ≥16 years and had a final diagnosis of a bacterial brain abscess (Table 1; Suppl Fig 1). All the data reported in this study are provided as a metadata table in .xlsx format (see link in supplementary data section). The majority of our cases were male (*n* = 36; 77%), in keeping with previous case series.^14^^,^^15^ The median age was 47 years (range 17–91).

**Table 1:** Demographic and host characteristics of a brain abscess cohort of 47 adults recruited in a UK tertiary referral hospital in the UK. sd=standard deviation; IQR=inter-quartile range.

Demographics	Male	Female	Total
Total number of patients (%)	36 (76.6)	11 (23.4)	47 (100)
Median age at presentation (sd)	46.6 (15.8)	63.0 (20.5)	46.9 (16.9)
Range of age at presentation (years) (IQR)	17–89 (38–59)	23–91 (41–64)	17–91 (38–61)
Mortality (%)	6/36 (16.7)	4/11 (36.4)	10/47 (21.3)
Risk Factors*:			
Intravenous Drug Use (%)	5/36 (13.9)	2/11 (18.2)	7/47 (14.9)
Immunosuppression⁎⁎(%)	1/36 (2.8)	1/11 (9.1)	2/47 (4.6)
Cardiac Anomaly (%)	7/36 (19.4)	1/11 (9.1)	8/47 (17.0)
Dental / ENT source (%)	10/36 (27.8)	4/11 (36.4)	14/47 (29.8)
No risk factor identified (%)	17/36 (47.2)	4/11 (36.4)	21/47 (44.7)

⁎ *Some individuals have > 1 risk factor(s).*

⁎⁎ *No patients were HIV positive (among n = 30 who received a test)*.

**Fig. 1:**
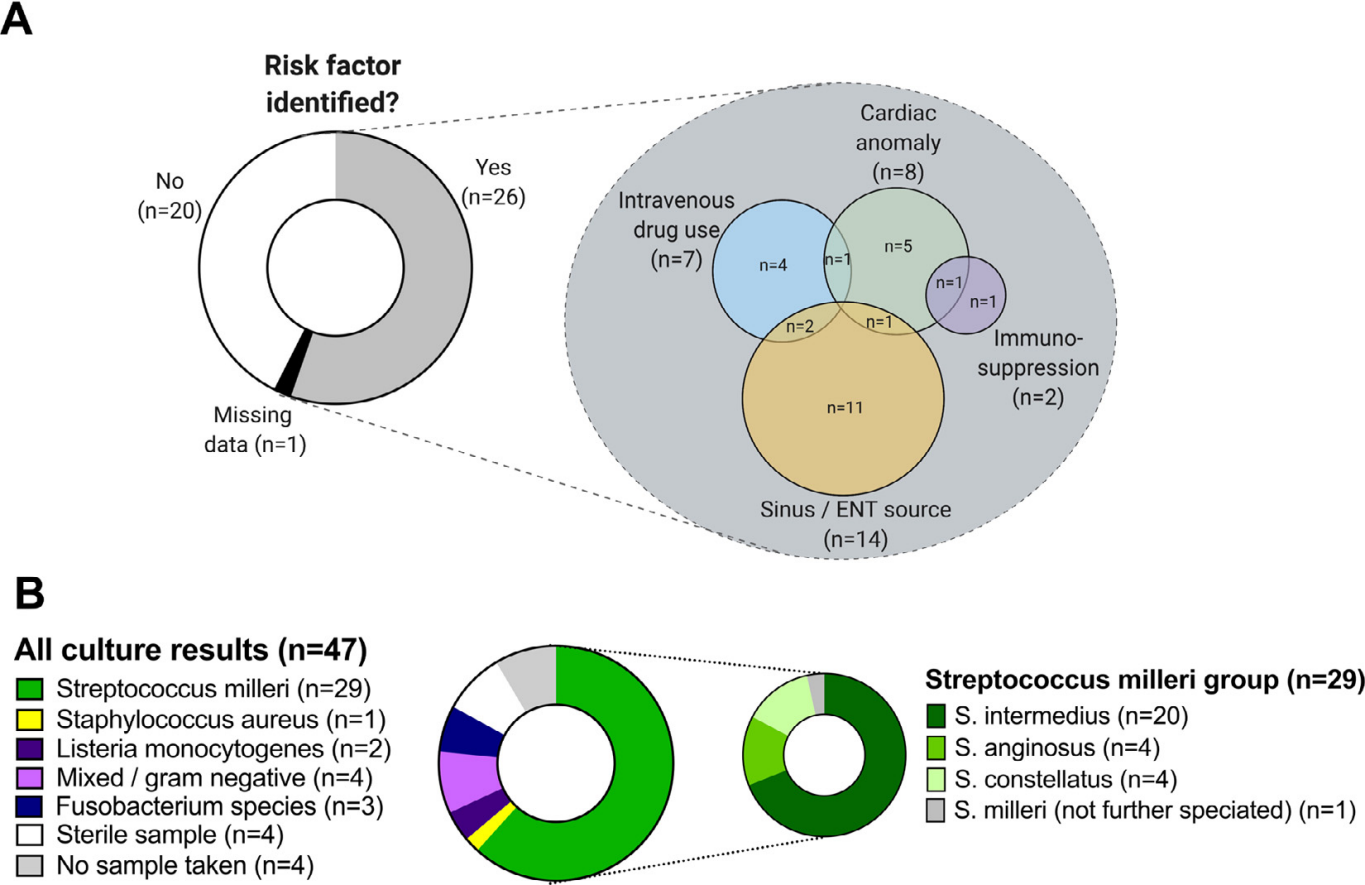
Summary of predisposing factors and underlying microbiology amongst 47 patients with brain abscess. A: Proportion of patients with and without predisposing factors, and a breakdown of the overlap between these factors. ENT = ears, nose and throat, including sinus infection. B: Culture results, showing predominance of *S. milleri* among all organisms, and predominance of *S. intermedius* within the *S. milleri* group.

**Fig. 2:**
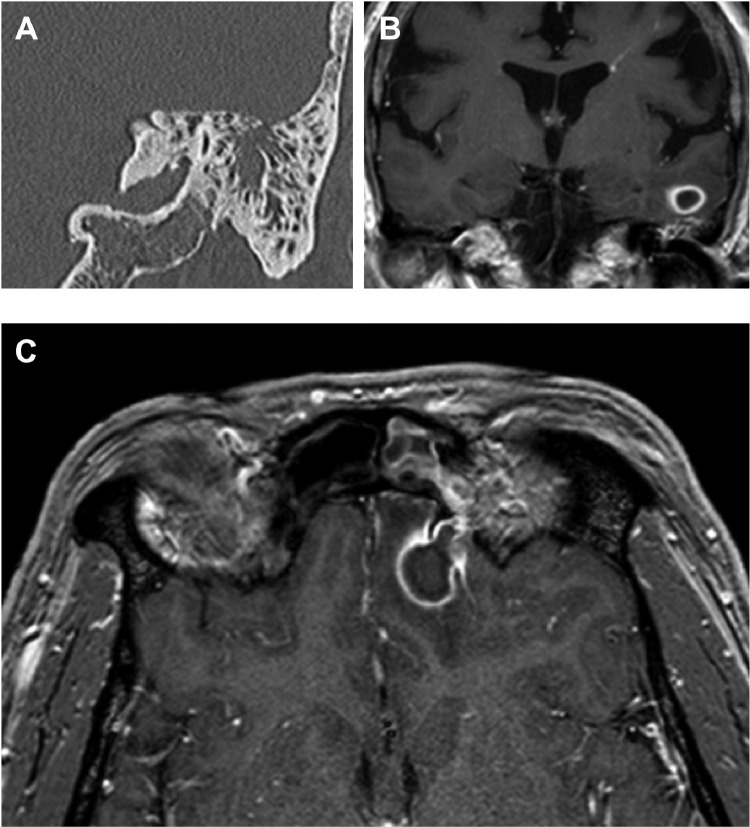
Neuro-imaging to demonstrate the anatomical relationship between sinus infection and brain abscess. A: Coronal high resolution CT image (shows left-sided otomastoiditis with focal breach of the bony roof of the mastoid. B: Coronal, gadolinium enhanced T1-weighted image, performed in the same patient 10 days after the CT scan, shows a small cerebral abscess inferiorly in the left temporal lobe adjacent to the infected mastoid (for panels A and B, patient ID: BA04). C: In a different patient, an axial, gadolinium enhanced T1-weighted image demonstrates a left frontal brain abscess secondary to adjacent left frontal sinusitis with focal breach of the posterior wall of the sinus (for panel C, patient ID: BA21).

An identifiable risk factor for brain abscesses was identified in 26 (55%; Fig 1A; Table 1). Sinus involvement was identified in 7/47 cases (15%; Table 2). Examples of the anatomical relationship between sinus infection and brain abscess are shown in Fig 2, and detailed radiological findings for all cases are included in the supplementary metadata table. HIV testing was not consistently undertaken, being reported for only 30/47 cases (64%), all of which were negative.

**Table 2:** Radiological features of brain abscesses at time of presentation.

Features of abscess	Number (%)
Number of abscesses	
Single	36/47 (77%)
Multiple	11/47 (23%)
Size of abscess	
Median cross-sectional size of abscess, mm^2^ (IQR)	500 (199–889)
Location of abscess(es):	
Frontal	15/47 (32%)
Parietal	8/47 (17%)
Temporal	7/47 (15%)
Occipital	4/47 (9%)
Cerebellum	2/47 (4%)
Other subcortical location	2/47 (4%)
Multiple locations	9/47 (19%)
Radiological features:	
Abscess wall enhancement	42/44* (95%)
Dual Rim Sign present	10/44* (23%)
Oedema present in one lobe	31/47 (66%)
Oedema present in >1 lobe	15/47 (32%)
Ventriculitis present	5/47 (11%)
Sinus involvement	7/47 (15%)

⁎ Difference in denominator due to three patients not receiving contrast enhancement.

### Brain abscesses were typically single lesions caused by streptococcus intermedius

The majority of abscesses were a single lesion (*n* = 36; 77%), commonest sites were frontal (*n* = 15; 32%) and parietal (*n* = 8; 17%); Table 2. Microbiological diagnosis was made in 39/47 cases (83%; Table 3), of which 34 were cultured from pus and five from blood cultures. Strikingly, organisms of the *S. milleri* group were present in 29/39 cases in which diagnostic data were available (74%), among which *S. intermedius* accounted for the majority (19/29; 66%; Fig 1B). In one case, *S. milleri* was identified from an orbital swab that had been collected in another clinical centre. Although the result of a swab should be interpreted with caution as potentially reflecting only colonising or commensal flora, in this case the growth of *S. milleri* is a plausible agent of deep-seated infection. Polymicrobial infection was identified in five cases, among which two involved organisms from the *S. milleri* group (Table 3). among the 29 *S. milleri* isolates, all were penicillin susceptible (confirmed by a recorded breakpoint in 18 cases, and by direct disc testing in 11; see supplementary metadata table for MIC values). Amongst the 6 anaerobic isolates, all were metronidazole sensitive.

**Table 3:** Pathogens identified in a cohort of 47 adults with a diagnosis of brain abscess.

Causative Organism	Number of cases (%)	Ceftriaxone Sensitivity
Gram positive infections		
Streptococcus intermedius^a^^,^^b^	20 (43%)	20/20 (100%)
Streptococcus constellatus^a^^,^^b^	4 (9%)	4/4 (100%)
Streptococcus anginosis^a^	4 (8.5%)	4/4 (100%)
Streptococcus milleri group (not further identified)^c^	1 (17.0%)	1/1 (100%)
Staphylococcus aureus^d^	1 (2.1%)	1/1 (100%)
Listeria monocytogenes^e^	2 (4.3%)	0/2 (0%)
Gram negative or mixed infections		
E. coli + Staphlyococcus epidermidis	1 (2.1%)	1/1 (100%)
Pseudomonas aeruginosa	1 (2.1%)	0/1 (0%)
Fusobacterium nucleatum^f^	3 (6.4%)	N/A
Citrobacter, Pseudomonas, Corynebacterium + anaerobes	1 (2.1%)	0/1 (0%)
Aggregatibacter aphrophilus + Actinomyces meyeri	1 (2.1%)	1/1 (100%)
No organism identified		
Sterile sample	4 (8.5%)	N/A
No sample taken	4 (8.5%)	N/A

a These organisms are all part of the *S. milleri* group.

b One *S. intermedius* and one *S. constellatus* reported in mixed culture with anaerobes.

c Isolate grown from an orbital swab.

d *S. aureus* sensitive to meticillin.

e Both patients with Listeria infection were >55 years of age but neither had any known cause of immunosuppression.

f Of the three cases with anaerobes isolated as a sole causative organism, two patients were intravenous drug users and the third had no clear identified source.

### Streptococcus intermedius was associated with larger abscesses

We investigated whether the presence of *S. milleri* was associated with any clinical or radiological characteristics and identified a significant relationship only with the size of the abscess (median cross-sectional size of abscess 930mm^3^ for *S. milleri* vs 190mm^3^ for other organisms; *p* = 0.0005; Suppl Table 1).

### Neurosurgical drainage was undertaken in the majority of cases

Among our 47 patients, 38 (81%) had an intervention to drain the abscess, and 15 underwent >1 drainage procedure (Suppl Table 2). The median time between admission for the brain abscess and first procedure (where first microbiological samples were taken) was 1 day (range 0–10 days). One individual had an elective biopsy for a presumed tumour. When the microbiology and histology suggested a diagnosis of a brain abscess, the patient was re-admitted for antibiotic treatment.

### Antibiotic treatment was undertaken for a median of six weeks

All patients were treated with intravenous antibiotics, predominantly using our local recommended first line antibiotic choice of ceftriaxone (used with or without metronidazole) in 39/47 cases (83%). Alternative choices justified on the grounds of clinical context and/or specific susceptibility results, with expert input based on review by our clinical infection/microbiology teams (specific prescriptions and rationale for choices are listed in Table 4). We did not identify any cases in which there was a deviation from local prescribing protocols. Duration of IV therapy was most commonly 6 weeks while follow-on oral therapy varied from 2–8 weeks (Suppl Fig 2).

**Table 4:** Intravenous and oral antibiotic regimens used to treat 46 adults with bacterial brain abscess. Data missing for one patient in cohort of 47.

Antibiotic agent(s)	Number (%)	Rationale for deviation from protocol for intravenous antibiotics (ceftriaxone)
Intravenous therapy		
Ceftriaxone monotherapy	5 (14.7%)	N/A
Ceftriaxone + metronidazole	34 (72.3%)	N/A
Amoxicillin + gentamicin	2 (4.3%)	L. monocytogenes infection
Ceftazidime monotherapy	1 (2.1%)	P. aeruginosa infection
Ceftazidime + metronidazole	1 (2.1%)	No organism identified, but healthcare acquired so risk of P. aeruginosa
Flucloxacillin	1 (2.1%)	Methicillin sensitive S. aureus infection
Meropenem monotherapy	1 (2.1%)	No organism identified, but patient immunosuppressed
Meropenem + vancomycin	1 (2.1%)	Mixed antibiotic-resistant organisms (potential AmpC-carrying Citrobacter species, Pseudomonas aeruginosa and penicillin resistant Corynebacterium)
ORAL THERAPY		
None	25 (53.2%)	N/A
Co-amoxiclav	7 (14.9%)	N/A
Amoxicillin	5 (10.6%)	N/A
Clindamycin	2 (4.3%)	N/A
Ciprofloxacin	2 (4.3%)	N/A
Died before oral switch	5 (10.6%)	N/A

N/A *= not applicable*.

### Brain abscesses are associated with high mortality

Ten patients in this cohort died (21%). Five (50%) deaths occurred before completion of the primary antimicrobial course and were directly attributable to the brain abscess (median 9 days after presentation; range 1–50 days), three were after the completion of treatment but showed possible attribution to brain abscess (median time post-presentation 116 days; range 76–146 days). One was attributed to unrelated comorbidities (113 days post-presentation), and one died at another centre with no further data available (146 days post-presentation). Of the 10 deaths, seven had significant medical comorbidities, including immunosuppression (*n* = 2), cardiac disease (*n* = 3), and other underlying medical conditions (*n* = 5).

Increasing age, immunosuppression, and the presence of an underlying cardiac anomaly were significantly associated with mortality on univariate analysis (*p* = 0.005, *p* = 0.04, *p* = 0.03, respectively; (Table 5). Mortality was not significantly associated with any radiological features or with any specific microbiological diagnosis.

**Table 5:** Associations between clinical features and mortality in a cohort of 47 adults with bacterial brain abscess.

Characteristic	Died (*n* = 10)	Survived (*n* = 37)	*p*-value^a^ (univariate)
Median Age (IQR)	60 (47 – 77)	46^38^^– 61^	0.005
Sex (proportion male)	6/10 (60%)	30/37 (81%)	0.16
Intravenous Drug Use	2/10 (20%)	5/37 (14%)	0.61
Cardiac Anomaly	4/10 (40%)	4/37 (11%)	0.03
Immunosuppressed^b^	2/10 (20%)	0/37 (0%)	0.04
Dental/ENT Source	3/10 (30%)	11/37 (30%)	0.99
Any risk factor present	8/10 (80%)	18/37 (49%)	0.15
Mean number of brain abscesses	1.89	1.35	<0.001
≥1 abscess present	4/10 (40%)	7/37 (19%)	0.21
Median cross-sectional size of abscess, mm^2^ (IQR)	707 (186–1272)	478 (236–865)	0.26
Aspiration/drainage undertaken	7/10 (70%)	31/37 (84%)	0.33
More than one aspiration/drainage procedure performed	1/10 (10%)	15/37 (41%)	0.13
Presence of microbiological diagnosis	7/10 (70%)	32/37 (86%)	0.22
Streptococcus milleri infection	6/10 (60%)	23/37 (62%)	1.0
Streptococcus intermedius infection	3/10 (30%)	16/27 (60%)	0.15
Oral antibiotics received^c^	0/5 (0%)	19/37 (51%)	0.05

a p-values for categorical variables calculated using Fisher's Exact test, for continuous variables using one-way ANOVA or Kruskal-Wallis, dependent on distribution of the data. Bold font indicates significant p value (<0.05).

b An HIV test was recorded for 30/47 cases (64%); all were negative. It is not clear from our data whether the remaining 17 were offered a test.

c Oral antibiotics refers to follow-on therapy after completion of a primary intra-venous course. Five patients died before completion of primary intravenous antibiotics.

## Discussion

### Microbiology

In this UK cohort of brain abscess patients, we found a low prevalence of Staphylococcal infection and a predominance of Streptococcal species, in particular *S. intermedius* of the *S. milleri* group. We identified a pathogen in 83% of cases, which is comparable to another recent UK case series ^7^ and compares favourably with two other data sets in which <60% of cases had a microbiological diagnosis.^14^^,^^15^ Improvements in sampling techniques, especially with relation to the timing of initial antibiotics, together with advances in microbiological laboratory practice (including the use of the MALDI-TOF) are likely reasons for the increased rates of laboratory diagnosis. Current expansion of metagenomic approaches to replace or supplement traditional phenotype-based methods ^27^^,^^28^ may underpin future improvements in identification rates.

The dominance of *S. milleri* species is consistent with other published UK epidemiological datasets on causative microbiology ^7^^,^^14^^,^^29^ and temporal trends in brain abscess epidemiology.^6^ The dominant role of *S. intermedius* is also exemplified by case reports of brain abscesses 30, 31, 32, 33, 34 and noted to be a frequent species in the *S. milleri* group isolated from central nervous system specimens.^35^ Another recent UK case series suggests the ‘*S. anginosus* group’ is predominant, but does not provide a breakdown of the *S. milleri* group by species.^7^ It is unclear how important speciation within the *S milleri* group is in clinical practice, given the entire group tends to be penicillin sensitive. *S. intermedius* may be dominant in brain abscesses as a result of its ecological niche in dental, sinus and ear infections.^36^ Its particular tendency to form abscesses may relate to the presence of specific virulence factors, including enzymes that digest host tissue (such as sialidase, hyaluronidase, and human-specific cytolysin),^37^ and factors that enhance binding to fibronectin and laminin in the extra-cellular matrix.^38^ This is in keeping with the significantly larger size of abscesses we found in association with these organisms.

Another striking finding from the microbiological data is the relative lack of staphylococcal infections. A recent systematic review reported staphylococci as still causing approximately 20% of brain abscesses,^6^ but *S. aureus* was found in <10% of cases in another recent UK series,^7^ and we identified only one case caused by *S. aureus*.

The small number of identified anaerobic infections (6/47, 12.8%) in our cohort is consistent with previous literature, including a systematic review reporting identification of anaerobes in approximately 10% of cases.^6^^,^^15^^,^^39^ This may be partly reflective of the difficulty in culturing anaerobes, particularly if sample transport to the laboratory is delayed. We did not collect specific data to determine whether any delays arose in this sample set, so were unable to investigate this point specifically. The accumulation of larger data sets of microbiological data based on molecular testing is needed to determine the contribution of anaerobes to intracranial abscesses.

### Antibiotic therapy

Our data demonstrate that ceftriaxone with metronidazole currently remains a safe empiric antimicrobial regimen in our setting.^40^ Narrower spectrum agents could be considered for the treatment of fully sensitive streptococci when supported by laboratory data but may be less easy to administer in one or two daily doses. Furthermore, culture-based methods may not accurately reflect the entire spectrum of organisms present, as evidence emerges for a complex microbiome within abscesses.^41^ A careful risk/benefit analysis is needed for inclusion of metronidazole on empiric grounds: an anaerobic contribution may be present even when not confirmed by laboratory testing, but addition of metronidazole can cause nausea, and prolonged therapy is associated with a risk of neuropathy.

Four to six weeks of therapy is typically suggested for intravenous ceftriaxone,^5^ but given the ongoing international drive to restrict and reduce the duration and use of broad spectrum antibiotics,^42^ it is important to consider whether typical prolonged parenteral antimicrobial regimens used in brain abscesses could potentially be restricted without loss of clinical efficacy, as is the case for other conditions, such as intra-abdominal infections,^43^ endocarditis ^22^ and osteomyelitis.^21^ Large randomised clinical trials with prospective follow-up are ideally needed to address the question about optimum route and duration of therapy, but in practice are difficult to conduct for a rare condition. The lack of guidance available on the choice, course and use of oral antibiotics ^16^^,^^20^ is reflected in our findings of a wide range of choices for follow-on antibiotics and their duration.

### Outcomes

Despite prompt surgical drainage and prolonged courses of antibiotic therapy, the mortality rate of this condition remains high, particularly in the context of frailty (in this setting, associated with immunocompromise, cardiac anomalies and older age). There was no increased mortality among patients in whom we did not secure a microbiological diagnosis, suggesting that empiric therapy is likely to be covering the underlying organisms present in this group. Five deaths occurred following completion of intravenous antibiotics in the group receiving no follow-on antibiotics; however, only one of these deaths was definitively attributable to brain abscess, while the other four were associated with significant co-morbidities (all had cardiac conditions and two were significantly immunosuppressed).

### Limitations

Our data rely upon the accuracy of diagnostic coding: just as some non-brain abscess cases were erroneously coded as G06.0, some genuine brain abscess cases may have been coded with a different ICD code. This relatively low incidence rate highlights the difficulties of performing interventional trials to examine conclusions further, and any such trial would need to be coordinated across multiple centres.

Our findings apply to a specific setting in the UK and should be extrapolated with caution. We recognise that the tertiary nature of our hospital may also bias the case-mix, either as a result of over-representing complex cases that need specialist multi-disciplinary management, and/or by the exclusion of certain groups – including those who are too unwell to transfer (with high mortality) and those with simple/limited disease who are managed in local hospitals without the need for referral to a specialist centre. We restricted the remit of our audit to adults, and we are therefore unable to extend our conclusions to brain abscesses in the paediatric population. Whilst our retrospective, observational approach adds important information that may help to underpin management decisions, there is a clear need for large prospective randomised controlled trials.

## Conclusion

From this study of the diagnosis and management of brain abscesses, we have provided contemporaneous UK epidemiological data in the context of limited published evidence. The predominant pathogen currently identified in this setting is penicillin-sensitive *S. intermedius,* but an emerging literature suggests a more complex microbiome. Multi-centre randomised prospective studies may be required to provide evidence underpinning the optimum choice, route and duration of antibiotic therapy.

## Appendix Supplementary materials

Image, application 1

Image, application 2
