# Supplementary material for: Microbial aetiology of brain abscess in a UK cohort: Prominent role of Streptococcus intermedius
Source: J Infect. 2020 Jun;80(6):623–9. doi: 10.1016/j.jinf.2020.03.011 (PMC7267774; doi:10.1016/j.jinf.2020.03.011)
Supplement: Supplementary file 1 [file mmc1.docx]

***SUPPLEMENTARY DATA***

**Suppl Figure 1: CONSORT diagram for identification of a cohort of adults with brain abscesses from a tertiary referral hospital in the UK.** G06.0 is the ICD code for ‘Intracranial abscess and granuloma’. VP=ventriculo-peritoneal.


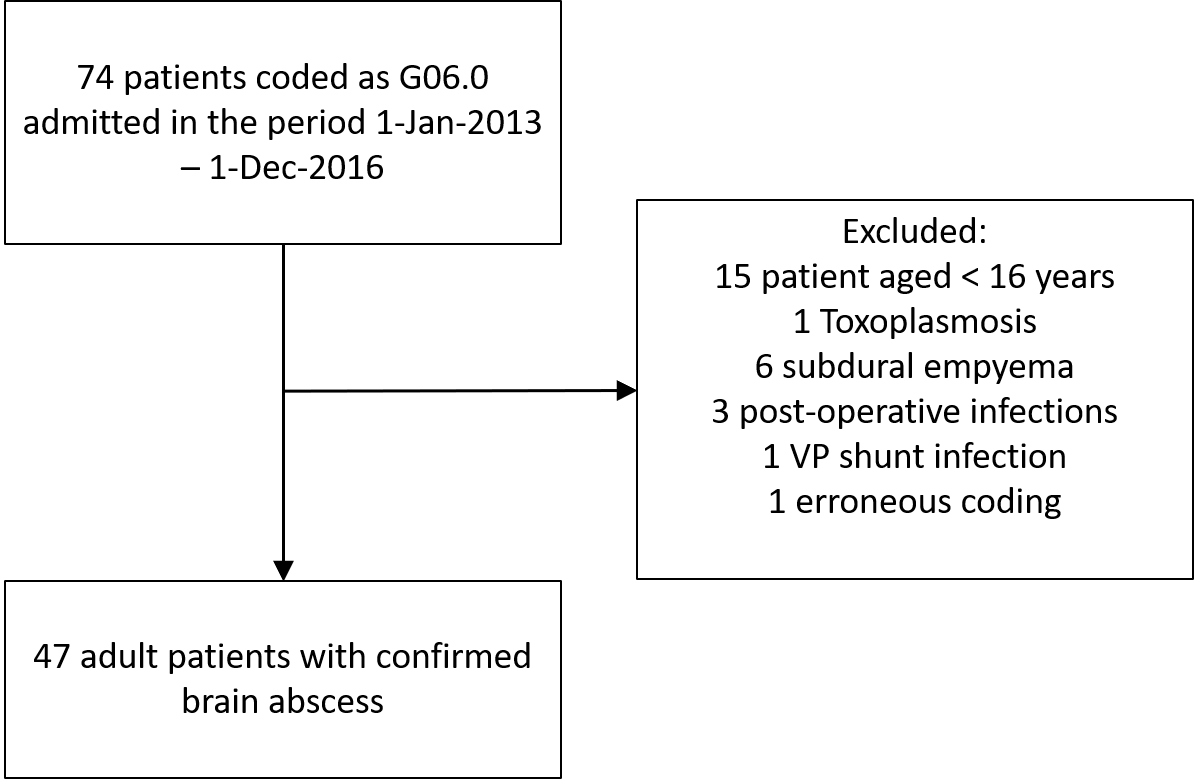


**Suppl Fig 2: Duration of antibiotic therapy for adults treated for bacterial brain abscess**

A: Duration of intra-venous ceftriaxone duration for 37 patients treated with this agent (two patients omitted due to death before course length could be decided). B: Duration of oral antibiotic follow-on (after primary intra-venous antibiotic course) for 19 patients.

| **A** | **B** |
| --- | --- |
| 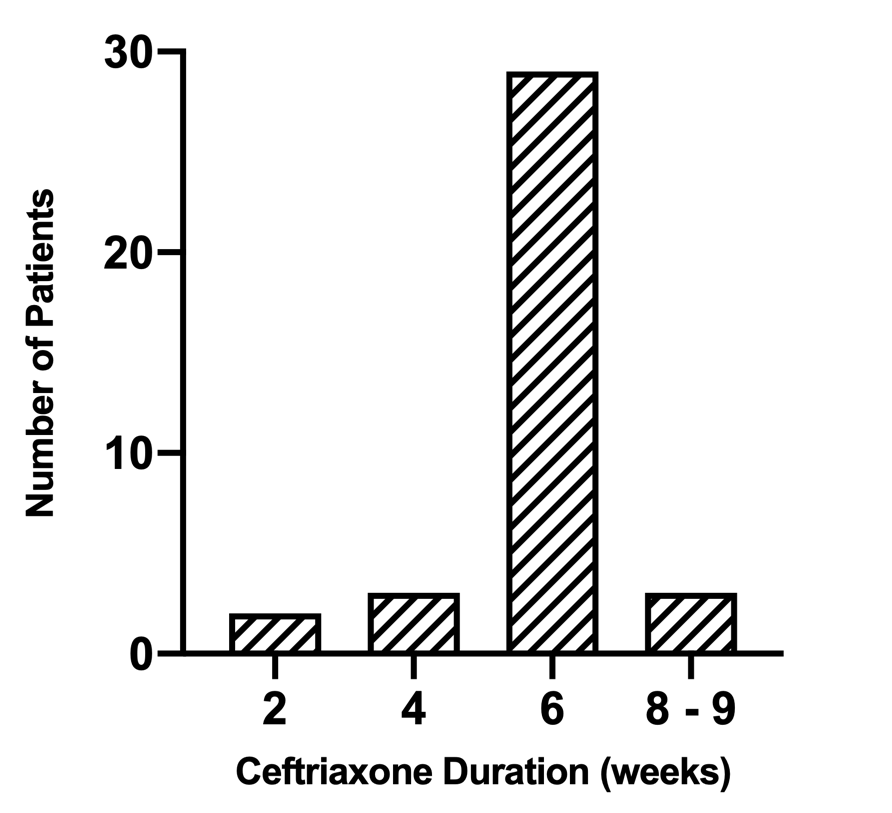 | 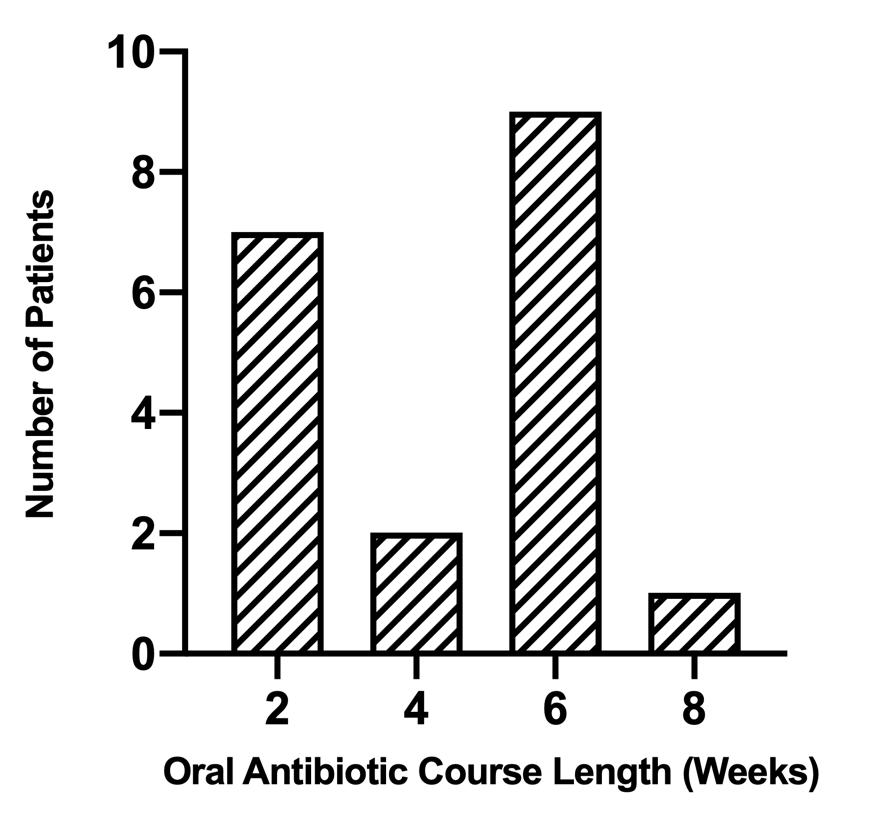 |

**Suppl Table 1: Association between *S. milleri* infection and other patient characteristics in a cohort of 39 adults with positive microbiology diagnosis of bacterial brain abscess** (8 patients in the cohort with no positive microbiology excluded from this analysis).

| **Characteristic** | **S. milleri**  **(n=29)** | **Non S. milleri**  **(n=10)** | **p-value^a^ (univariate)** |
| --- | --- | --- | --- |
| Median Age (IQR) | 48 (40-61) | 43 (34-62) | 0.6 |
| Sex (proportion male) | 24/29 (83%) | 8/10 (80%) | 1.0 |
| Intravenous Drug Use | 4/29 (14%) | 3/10 (30%) | 0.3 |
| Cardiac Anomaly | 5/29 (17%) | 2/10 (20%) | 1.0 |
| Immunosuppressed | 0/29 (0%) | 0/10 (0%) | N/A |
| Dental/ENT Source | 8/29 (28%) | 3/10 (30%) | 1.0 |
| More than one abscess | 7/29 (24%) | 2/10 (20%) | 0.4 |
| Median cross-sectional size of abscess, mm^2^ (IQR) | 730 (478-1107) | 149 (79-312.) | **0.01** |
| Died | 6/29 (21%) | 1/10 (10%) | 0.7 |

*^a^ Bold font indicates significant p value (<0.05)*

**Suppl Table 2: Number of surgical interventions undertaken for 47 adults presenting with brain abscess**

| Number of Neurosurgical Interventions | Number of patients | Mortality | Median age (range) |
| --- | --- | --- | --- |
| None | 9 (19.1%) | 3/9 (33.0%) | 56.8 (23.1 – 82.1) |
| One | 21 (44.7%) | 5/21 (23.8%) | 46.9 (22.5 – 91.5) |
| Two | 12 (25.5%) | 1/12 (8.3%) | 46.2 (23.9 – 65.0) |
| Three | 4 (8.5%) | 0/4 (0%) | 40.4 (17.4 – 50.6) |
| Four | 1 (2.1%) | 0/1 (0%) | 61.2 |
