# Supplementary material for: Microbial aetiology of brain abscess in a UK cohort: Prominent role of Streptococcus intermedius
Source: J Infect. 2020 Jun;80(6):623–9. doi: 10.1016/j.jinf.2020.03.011 (PMC7267774; doi:10.1016/j.jinf.2020.03.011)
Supplement: Supplementary file 2 [file mmc2.docx]

**SUPPLEMENTARY DATA**

All data generated or analysed during this study are included in this published article, and its Supplementary Information files, which are accessible on-line at Figshare: <https://doi.org/10.6084/m9.figshare.11662950.v1>.

Supplementary on-line files comprise the following:

- **Metadata table: xls spreadsheet containing full dataset of clinical, radiological and imaging data presented in this study.**
- **STROBE statement (Strengthening the Reporting of Observational Studies in Epidemiology)**
- **File containing suppl figures and tables:**
  - **Suppl Fig 1:** CONSORT diagram for identification of a cohort of adults with brain abscesses from a tertiary referral hospital in the UK.
  - **Suppl Fig 2:** Duration of antibiotic therapy for adults treated for bacterial brain abscess
  - **Suppl Table 1:** Association between *S. milleri* infection and other patient characteristics in a cohort of 39 adults with positive microbiology diagnosis of bacterial brain abscess
  - **Suppl Table 2:** Number of surgical interventions undertaken for 47 adults presenting with brain abscess
